# Three metabolic pathways are responsible for the accumulation and maintenance of high AsA content in kiwifruit (*Actinidia eriantha*)

**DOI:** 10.1186/s12864-020-07311-5

**Published:** 2021-01-06

**Authors:** Guanglian Liao, Lu Chen, Yanqun He, Xishi Li, Zhengxin Lv, Shuyao Yi, Min Zhong, Chunhui Huang, Dongfeng Jia, Xueyan Qu, Xiaobiao Xu

**Affiliations:** 1 College of Forestry, Jiangxi Agricultural University/Jiangxi Provincial Key Laboratory of Silviculture, Nanchang, Jiangxi 330045 PR China; 2 College of Agronomy, Jiangxi Agricultural University/Kiwifruit institute of Jiangxi Agricultural University, Nanchang, Jiangxi 330045 PR China; 3 Jinggangshan Institute of Biotechnology, Jinggangshan Branch of Jiangxi Academy of Sciences, Ji’an, Jiangxi 343,016 PR China

**Keywords:** Ascorbic acid, Enzyme activity, Gene expression, Transcriptomics

## Abstract

**Background:**

*Actinidia eriantha* is a precious material to study the metabolism and regulation of ascorbic acid (AsA) because of its high AsA content. Although the pathway of AsA biosynthesis in kiwifruit has been identified, the mechanism of AsA metabolism and regulation is still unclear. The purpose of this experiment is to reveal the AsA metabolic characteristics of *A. eriantha* ‘Ganmi 6’ from the molecular level, and lay a theoretical foundation for the research on the genetic improvement of kiwifruit quality.

**Results:**

We found that AsA reached the accumulation peak at S7 (110 DAF) during the process of fruit growth and development. The activity of GalDH, GalLDH, MDHAR and DHAR in fruit was similar to AsA accumulation trend, and both of them were significantly positively correlated with AsA content. It was speculated that GalDH and GalLDH were key enzymes in AsA biosynthesis, while MDHAR and DHAR were key enzymes in AsA regeneration cycle, which together regulated AsA accumulation in fruit. Also, we identified 98,656 unigenes with an average length of 932 bp from the transcriptome libraries using RNA-seq technology after data assembly. There were 50,184 (50.87%) unigenes annotations in four databases. Two thousand nine hundred forty-nine unigenes were enriched into the biosynthesis pathway of secondary metabolites, among which 133 unigenes involved in the AsA and aldehyde metabolism pathways, and 23 candidate genes related to AsA biosynthesis, cycling and degradation were screened out.

**Conclusions:**

Considering gene expression levels and changes of physiological traits and related enzyme activity, we concluded that the accumulation of AsA depends mainly on the L-galactose pathway, and the D-galacturonic acid pathway and AsA recycling pathway as the secondary pathways, which co-maintain the high AsA content in fruit of *A. eriantha*.

**Supplementary Information:**

The online version contains supplementary material available at 10.1186/s12864-020-07311-5.

## Background

Ascorbic acid (AsA) is one of the antioxidants abundant in plant tissues. It is involves in plant cell oxidation, photosynthesis protection, cell division, growth and signal transduction, and plays a crucial role in plant development and abiotic stress tolerance [1]. With further research, the role of AsA in plants and humans may have gone far beyond traditional understanding. As known for people, kiwifruit have high nutrient content, e.g., the content of AsA in the fruit is 3 or 4 times than that in apple or other fruit [2]. Kiwifruit is an excellent material for studying the metabolism of AsA.

**Fig. 1:**
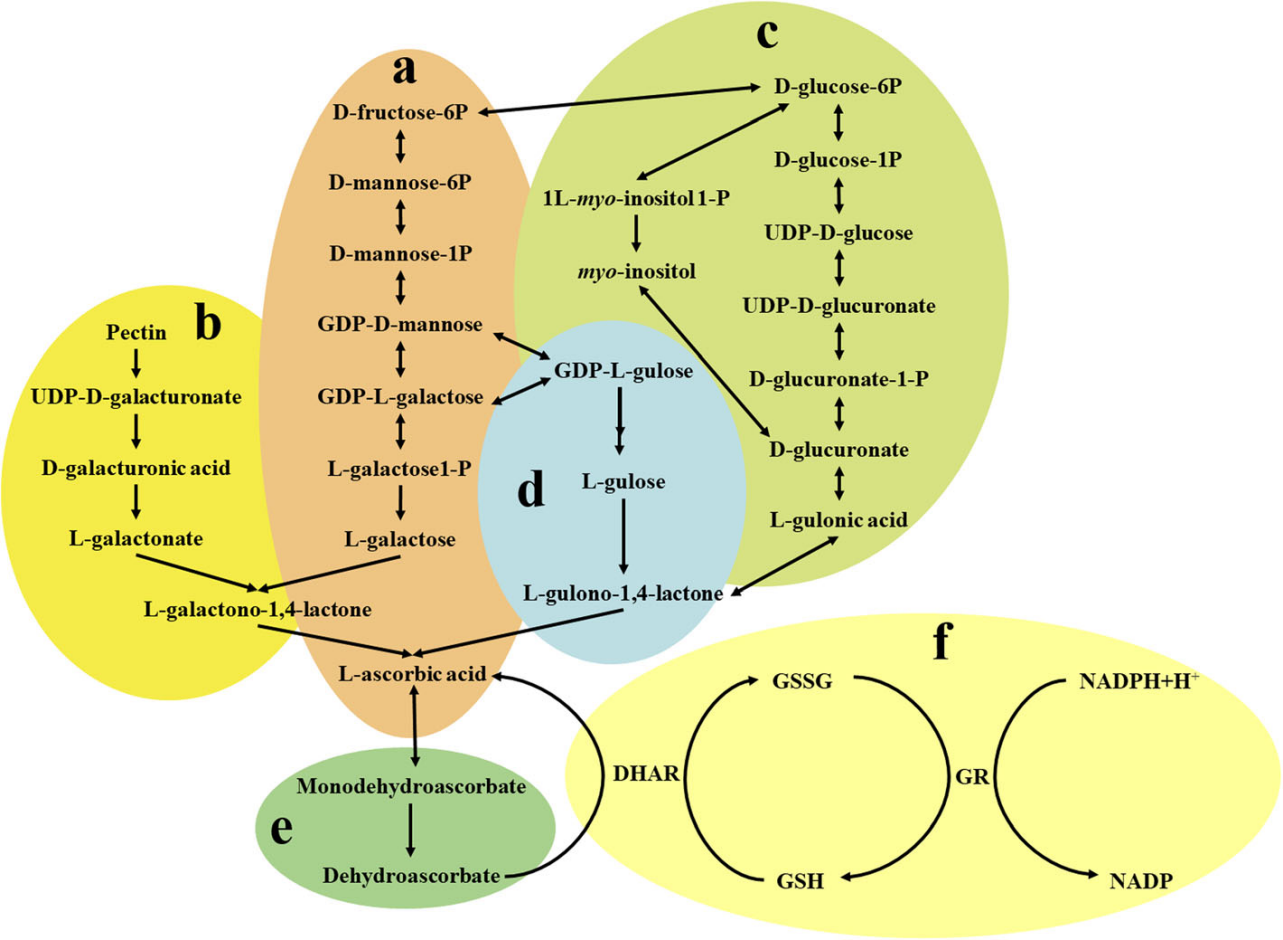
Synthesis and degradation of L-ascorbic acid: **a**, L-galactose pathway; **b**, D-galacturonic acid pathway; **c**, inositol pathway; **d**., L-gulose pathway; **e**, AsA degradation pathway; **f**, AsA-GSH circulation pathway [3–6]. DHAR, dehydroascorbate reductase; GSSG, oxidized glutathione; GSH, reduced glutathione; GR, glutathione reductase; NADP, nicotinamide adenine dinucleotide phosphate; NADPH, the reduced form of nicotinamide adenine dinucleotide [6]

In the process of plant growth, AsA accumulation is mainly regulated by biosynthesis, cycling and degradation. So far, the L-galactose pathway (Fig. 1a), L-gulose pathway (Fig. 1d), D-galacturonic acid pathway (Fig. 1b) and the inositol pathway (Fig. 1c) are currently recognized AsA biosynthesis pathways [3–5]. In addition, the ascorbic acid-glutathione cycle (ASA-GSH) [6] (Fig. 1f) is also an effective balance between biosynthesis, oxidation (Fig. 1e), and cycling in plants. Not only structural genes but also many factors (such as light, temperature, ozone, hormones and regulatory factors) can also regulate the accumulation of AsA [7].

In the L-galactose pathway, the D-glucose-6-phosphate molecule is converted to D-fructose-6-phosphate in the presence of glucose isomerase (PGI) [8]. It was then converted to D-mannose − 6-phosphate and D-mannose − 1-phosphate by phosphomannose isomerase (PMI) [9] and phosphoric mannose enzyme (PMM) [10] catalysis. GDP-D-mannose-3 ‘and 5’ -hetero isomerase (GME) located downstream of GDP-mannose pyrophosphorylase (GMP) and converts GDP-D-mannose-3 ‘to GDP-L-galactose, which is considered as the central enzyme in AsA biosynthesis [11]. Overexpression of *GMEs* (*SlGME1* and *SlGME2*) in transgenic tomato plants increased the AsA concentration, but the expression of *GME* in kiwifruit could not determine the AsA level, and similar conclusions were obtained in peach [12]. L-galactose is oxidized by L-galactose dehydrogenase (GalDH) to L-galactose-1, 4-lactone, a direct substrate of AsA bioscience. Overexpression of *GalDH* in tobacco increased the activity of GalDH, but did not increase the AsA content in leaves, the expression level of *GalDH* in apples was not significantly correlated with the AsA content [13]. However, the opposite results were obtained in kiwifruit, navel orange and roxberry [14]. L-galactono-1,4-lactone dehydrogenase (GalLDH) is a key enzyme in the last step of l-galactose biosynthesis pathway in plant AsA and catalyzes the production of AsA by L-galactose-1, 4-lactone. GalLDH has high specificity and conversion efficiency for L-galactose-1, 4-lactone. Reducing cytochrome C, as an electron receptor in the respiratory chain [15], can rapidly convert exogenous L-galactose-1, 4-lactone into AsA.

The D-galacturonic acid pathway is an alternative to the AsA biosynthetic pathway. D-galacturonic acid is reduced to D-galacturonic acid under the action of D-galacturonic acid reductase (GalUR), and then L-galacturonic acid 1, 4-lactone are formed under the action of aldolactase. At this time, L-galacturonic acid is merged with the L-galacturonic acid pathway and directly oxidized to AsA under the action of *GalLDH*. The expression level of *GalUR* in *A. deliciosa* was highly consistent with that of AsA [16]. The L-gulonic pathway converts glucose to D-glucuronic acid, and inositol oxygenase (MIOX) converts inositol to D-glucuronic acid. Conversion of inositol to D-glucuronic acid by MIOX may potentially serve AsA substrate. Then, D-glucuronic acid is converted into L-gullose-1, 4-lactone by a series of enzymes, which then enters the L-gullose pathway and is converted into AsA under the action of L-gulose.

In plants, AsA content is also highly regulated by the regeneration and recycling system, which is an important way to oxidative AsA regeneration. L-ascorbate oxidase (AAO) and L-ascorbate peroxidase (APX) are key enzymes in the antioxidant system of plants, as well as key enzymes in scavenging free radicals in the body. APX uses AsA as a specific electron donor to catalyze the conversion of H_2_O_2_ into H_2_O and O_2_, and AsA is oxidized to MDHA. Part of MDHA is reduced to AsA through the catalytic action of MDHAR, while part of MDHA is converted to DHA through non-enzymatic disproportionation, and DHA is reduced to AsA through the joint participation of DHAR and glutathione (GSH). After *MDHAR* was overexpressed in tomato ‘Micro-Tom’, the AsA content of fruit was increased by 1.7 times. After increasing the expression level of *DHAR*, the AsA content of fruit was increased by 1.6 times [17].

It is of great significance to study the gene function of *A. eriantha* and to develop and utilize the germplasm resources through RNA-Seq technology to establish a platform for the kiwifruit functional gene research data. At the same time, combined with the changes in related physiological indicators for comprehensive analysis, the study of the gene expression in the metabolic pathways can initially reveal the molecular mechanism of fruit development of *A. eriantha*, and would lay an important scientific basis for kiwifruit breeding, germplasm innovation and variety improvement.

## Results

### Kiwifruit development

**Fig. 2:**
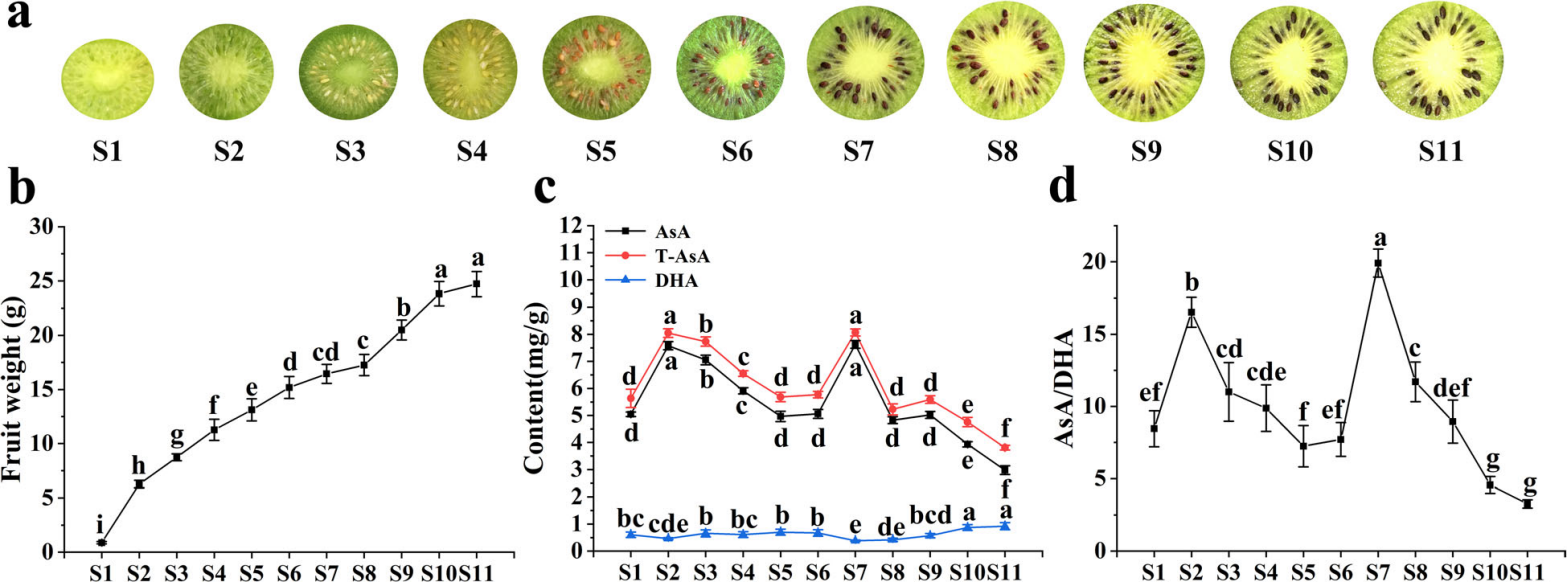
Fruit development of ‘Ganmi 6’. Fruit: 20 DAF (S1), 35 DAF (S2), 50 DAF (S3), 65 DAF (S4), 80 DAF (S5), 95 DAF (S6), 110 DAF (S7), 125 DAF (S8), 140 DAF (S9), 155 DAF (S10) and 170 DAF (S11). **a** The transverse of ‘Ganmi 6’ during the fruit development. **b** The fruit weight change of ‘Ganmi 6’ during the fruit development. **c** Changes of AsA, T-AsA and DHA content of ‘Ganmi 6’ during the fruit development. **d** Changes of AsA/DHA ratio during the fruit development. Duncan’s method was used to detect the differences between different development of fruit at *P ≤ 0.05*. In the same index, there was no significant difference between stages with the same lowercase letter

The ‘Ganmi 6’ kiwifruit samples used as the test materials were selected at eleven developmental stages from initial fruit appearance at 20 DAF to its full maturity at 170 DAF (Fig. 2a). S1-S6 is the first rapid growth stage of fruit (Fig. 2b) and the seeds have been brown (Fig. 2a), S8-S10 is the second rapid growth stage of fruit (Fig. 2b). AsA content was also determined, it was found that AsA began to accumulate and its content increased at the early stage of fruit development, reached the first peak of AsA accumulation at S2 (7.56 mg·g^− 1^). Subsequently, AsA content continued to decline until S7, the decline was interrupted and reached the second peak (7.63 mg·g^− 1^). AsA content resumed its downward trend at S8 and has a steep decrease at S9, then decreased until it’s harvested (Fig. 2c). The dynamic changes of T-AsA content and AsA/DHA (Fig. 2d) was basically consistent with that of AsA content, the DHA content were lower throughout development (Fig. 2c).

### Activity of AsA related metabolic enzymes

**Fig. 3:**
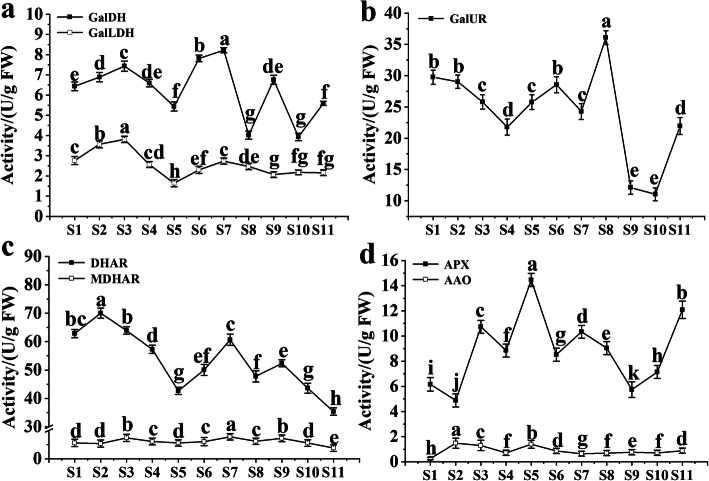
Dynamic changes in the activities of AsA related metabolic enzymes activities of different pathway, included L-galactose pathway (**a**), D-galacturonic acid pathway (**b**), AsA regeneration pathway (**c**) and AsA decomposition pathway (**d**). FW: fleshed weight. Duncan’s method was used to detect the differences between different development of fruit at *P ≤ 0.05*. In the same enzyme, there was no significant difference between stages with the same lowercase letter

The activity of dehydroascorbate reductase (DHAR) was higher than that of other enzymes in the whole development period, between 35.38–70.04 U·g^− 1^ FW, and the first and second peak of DHAR activity coincided with the peak of AsA (Fig. 3c). D-galacturonic acid reductase (GalUR) activity was between 11.04–36.05 U·g^− 1^ FW, reached the maximum value (36.05 U·g^− 1^ FW) at S8, and then declined rapidly until GalUR activity reached the minimum value (11.04 U·g^− 1^ FW) at S10 (Fig. 3b). The lowest activity of ascorbic peroxidase (APX) corresponds to the first peak of AsA, and with the increase of ascorbic acid content, the activity of APX decreases during S2-S5, with the ripening of fruit, AsA content decreased and APX activity increased (Fig. 3d). The activity trend of L-galactose dehydrogenase (GalDH, Fig. 3a) and L-galactono-1,4-lactone dehydrogenase (GalLDH, Fig. 3a) was similar to that of AsA content, the range was 3.76–8.21 and 1.65–3.81, respectively. The changes of monodehydroascorbate reductase (MDHAR, Fig. 3c) and L-ascorbate oxidase (AAO, Fig. 3d) activities were relatively small during fruit development, were 3.75–7.73 and 0.26–1.39 respectively. In addition, there was a similar trend between the MDHAR and AsA.

### Sequencing, basic transcriptome assembly, and functional annotation

We obtained 98,565 unigenes with an average length of 932 bp, the longest unigenes sequence length is 16,709 bp, the shortest unigenes sequence length is 201 bp, and N50 of 1609 bp (supplementary Table 2). The length distribution of unigenes (supplementary Figure 1) showed that the number of genes decreased gradually with the increase of gene length.

The 98,656 unigenes obtained by the assembly were aligned to Nr, SwissProt, KOG and KEGG databases. A total of 50,184 unigenes were annotated, with an annotation rate of 50.87% (supplementary Figure 2).

Of the 98,656 unigenes, 88,867 (90.08%) were annotated with GO terms that were offered by the GO-based annotation (an internationally standardized gene functional classification system) as a strictly defined conceptualization for comprehensively describing the properties of genes and their products within any organism. The 88,867 unigenes were classified into three functional categories: biological process, cellular component, and molecular function (supplementary Figure 3).

A total of 28,919 unigenes were annotated in the KOG database, and these unigenes were divided into 25 categories. Among them, general function prediction only (R), posttranslational modification (O) and signal transduction mechanisms (T) were the dominant categories (supplementary Figure 4).

### KEGG pathway analysis

A total of 31,069 unigenes were annotated into the KEGG database and assigned to the following five KEGG biochemical pathways (supplementary Figure 5). We pay more attention to AsA and aldehydic acid pathway, which enriched 133 unigenes, these unigenes were involved in ASA metabolism.

### Screening of DEGs

The focus of the present study is that the decreasing trend of AsA content was interrupted at S7. Three group comparisons consisting of S6 vs S7, S6 vs S8, S7 vs S8 were determined to identify the DEGs. As can be seen from supplementary Figure 6, the down-regulated genes were more than the up-regulated genes in the three periods of S6, S7 and S8. AsA content in fruit depends on its biosynthesis ability and degradation recycling level. AsA biosynthesis and degradation are catalyzed by a series of enzymes. The level of gene expression that encodes these enzymes directly determines the amount of AsA synthesis or degradation. Finally, according to the DEGs among S6 vs S7, S6 vs S8 and S7 vs S8, the 23 unigenes related to AsA anabolism and with high expression were screened out (supplementary Table 3). These unigenes are mainly distributed in the AsA biosynthetic pathway including L-galactose pathway, D-galacturonic acid pathway and inositol pathway as well as the AsA circulation pathway.

### Expression profiles of 23 DEGs, cluster analysis and correlation analysis

**Fig. 4:**
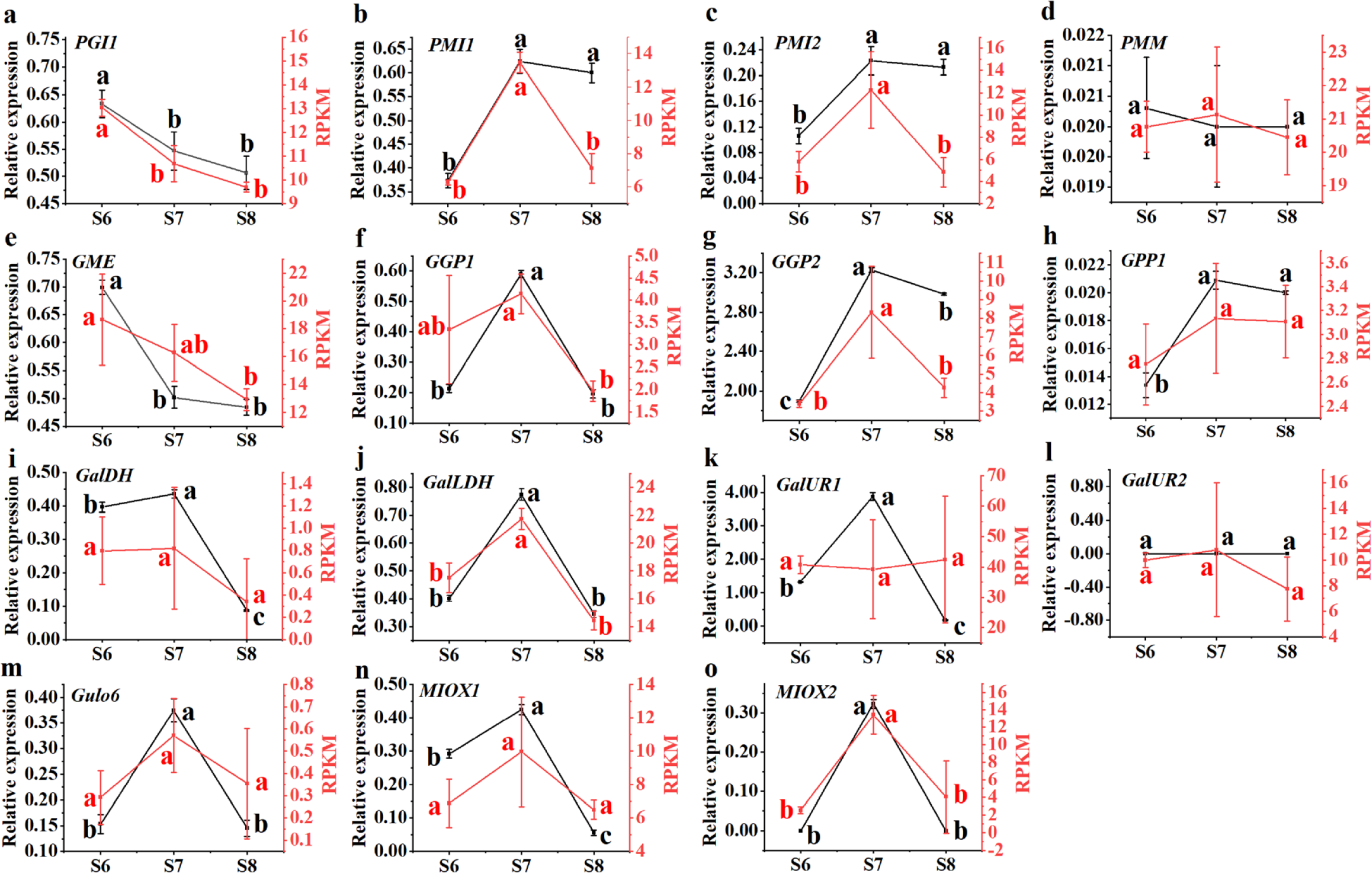
Expression analysis of genes related to the AsA synthesis pathway. L-galactose pathway: **a**-**j**, D-galacturonic acid pathway: g and h, L-gullosugar pathway: m, inositol pathway: **n** and **o**. Duncan’s method was used to detect the differences between different development of fruit at *P ≤ 0.05*. In the same type of data, there was no significant difference between stages with the same lowercase letter

The qRT-PCR data of 23 candidate genes were compared with transcriptome data, we found that the trends were basically the same. The gene expression involved in the AsA synthesis pathway was shown in Fig. 4, in the L-galactose pathway, *PGI1* expression was higher in early and late fruit development (supplementary Figure 7 & Fig. 4a); the relative expression level of *PMI1* was higher than that of *PMI2* throughout development (Fig. 4b and c); the relative expression level of *PMM* and *GPP1* was similar and generally low throughout development (Fig. 4d and h); the relative expression level of *GME* was higher in the early stage of fruit development and showed a downward trend as a whole (Fig. 4e); the relative expression level of *GGP2* is higher than that of *GGP1*, and the relative expression level of *GGP1* generally shows a downward trend (Fig. 4f and g); the expression trends of *GalDH* and *GalLDH* were basically similar, generally showing a descending - then ascending - and then descending trend, and the expression levels of both were higher in early fruit development (Fig. 4i and j). As for the D-galacturonic acid pathway, the relative expression level of *GalUR1* was higher than that of *GalUR2* in the whole development stage, the relative expression level of *GalUR1* is the highest at S7, and the relative expression level of *GalUR2* was almost undetectable after S3 (Fig. 4k and l). L-gulonolactone oxidase gene (*GuLO*) is the structural gene in the last step of AsA synthesis in L-gullosugar pathway, the expression of *GuLO6* shows a downward trend during the whole development period, and the expression level of *GuLO6* was significantly decreased after S7 (Fig 4m). Finally, the relative expression levels of *MIOX1* and *MIOX2* in the inositol pathway were low (Fig. 4n and o).

**Fig. 5:**
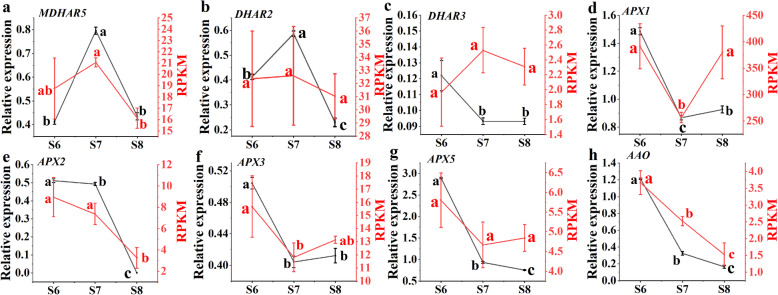
Expression analysis of genes related to the AsA cycle pathway. Duncan’s method was used to detect the differences of different development of fruit at *P ≤ 0.05*. In the same type of data, there was no significant difference between stages with the same lowercase letter

In AsA cycle pathway, the expression of *MDHAR* fluctuated in early fruit development and decreased in late fruit maturity (supplementary Figure 8 & Fig. 5a); the relative expression of *DHAR2* showed an overall downward trend, and increased at S7(Fig. 5b); the relative expression of *DHAR3* was lower during the whole fruit development stage (Fig. 5c); the relative expression levels of *APX1* and *APX5* were consistent, lower in the early and late-stage and higher in the middle stage of fruit development (Fig. 5d and g); the relative expression levels of *APX2*, *APX3* and *AAO* showed a downward trend, in which the relative expression levels of *AAO* could hardly be detected in the later stage of fruit development (Fig. 5e, f and h).

### Correlation analysis of metabolism components, related enzymes and genes of AsA

It can be seen from supplementary Table 4, AsA content in fruit was significantly negatively correlated with DHA content (R^2^ = − 0.65), and was significantly positively correlated with T-ASA (R^2^ = 1.00) and AsA/DHA (R^2^ = 0.89), respectively; DHA content was significantly negatively correlated with T-ASA (R^2^ = − 0.59) and AsA/DHA (R^2^ = − 0.85), respectively; and T-ASA and AsA/DHA (R^2^ = 0.85) were significantly positively correlated.

GalDH and GalLDH in the synthetic pathway and MDHAR and DHAR in the regenerative cycle pathway were significantly correlated with AsA content, and the correlation coefficients were 0.73, 0.73, 0.68 and 0.89, respectively. DHA was negatively correlated with the activity of MDHAR (R^2^ = − 0.61) and DHAR (R^2^ = − 0.60). The activity of T-ASA was significantly positively correlated with GalDH (R^2^ = 0.74), GalLDH (R^2^ = 0.74) and DHAR (R^2^ = 0.88), while the activity of MDHAR (R^2^ = 0.66) was significantly positively correlated with T-ASA.

*GME*, *GGP1* and *GalLDH* in L-galactose pathway were significantly correlated with the expression levels of *GalUR1* in D-galacturaldehyde pathway and *MDHAR5*, *DHAR2* and *AAO* in AsA regeneration cycle pathway, with correlation coefficients of 0.67, 0.61, 0.58, 0.87, 0.63, 0.60 and 0.59, respectively.

## Discussion

AsA is one of the important indexes of kiwifruit quality evaluation. AsA content varies greatly among different species, even for kiwifruit of different species belonging to *Actinidia* genus, such as *A. eriantha* and *A. chinensis*, AsA content of ripening fruit is also very different. In addition to its own factors, AsA content in plants is also influenced by environmental factors and management level, including lighting, altitude, relative humidity, temperature, orchard fertilizer and water management, pruning and other factors [18]. The changes of AsA content in this study were basically consistent with previous studies [19], however, it is worth noting that when AsA has a rising peak, the seeds just change from brown to black. We hypothesized that AsA metabolism might be related to seed development, which should be further study.

*GME* is considered to be a key regulator gene of the synthesis of AsA by the L-galactose and L-gullosugar pathways [11]. After the expression of *GME* was silenced, AsA content in tomatoes was significantly reduced, which seriously impaired the growth and development of plants [20]. At the same time, AsA accumulation was increased and abiotic stress tolerance was significantly enhanced in tomatoes after *GME* overexpressed [12]. However, for the early studies of kiwifruit [21], only silences or overexpressed the *GME* may not determine the AsA level. In this study, the relative expression of *GME* was relatively high during the fruit development period, but decreased as the fruit ripened, and there was a significant correlation between the relative expression of *GME* and AsA content, indicating that *GME* affects the accumulation of AsA in fruit to some extent. However, it is not an important regulatory gene for the brief peak of AsA in this study. As for GalDH, which is considered as one of the key rate-limiting enzymes in the L-galactose pathway for AsA synthesis, the expression level of GalDH was closely related to AsA content [14]. In this study, the activity of GalDH was significantly positively correlated with the AsA content, which was consistent with the results of previous studies [22], indicating that GalDH is one of the key rate-limiting enzymes in the L-galactose pathway in *A. eriantha*. GalLDH is the last key enzyme in the synthesis of AsA in L-galactose pathway, and its activity and transcriptional level directly determine the AsA content. Many studies have shown that the increase of ascorbic acid content can be achieved by increasing the expression of *GalLDH*, such as in apple [13], rice [23] and potato [24]. Studies on kiwifruit showed that GalLDH activity in mature fuit of different genotypes was significantly correlated with T-AsA and AsA [25]. Genes (high expression) and key enzymes (high activity) in the L-galactose pathway that are obviously related to AsA content may be important factors for maintaining high AsA content in fruit, but they are not necessarily the only factors. It can be speculated that the L-galactose pathway is the main way for AsA synthesis in the fruit of *A. eriantha* ‘Ganmi 6’.

In this study, we also found several other genes involved in the synthesis of AsA. *GalUR* is a key regulatory gene of D-galacturonic acid pathway. Many studies have shown that GalUR plays an important role in AsA synthesis in many plants. After the overexpression of *GalUR* in *Arabidopsis thaliana*, AsA content increased by 2–3 times [26]. In addition, the AsA content of strawberry fruit at the mature stage were mainly controlled by the D-galacturonic acid pathway, and with the increase of AsA content and the increase of *GalUR* expression in fruit [27]. In this study, AsA content was significantly correlated with the expression level of GalUR1, but there was little correlation between AsA content and the activity of GalUR, which means that D-galacturonic acid pathway is not the main pathway of AsA synthesis, but an important auxiliary pathway. The L-gullosugar and inositol pathways are more common in animals, and these two synthetic pathways are initiated in plants under special circumstances. In this study, there was no significant correlation between the relative expression trend of *GuLO6* and *MIOX* and AsA content. This means that the inositol pathway and the L-coulomb pathway have little influence on the AsA content in the fruit of *A. eriantha* or that the inositol pathway makes little contribution to the synthesis of AsA under normal conditions.

AsA regeneration cycle pathway also plays an important role in AsA accumulation. Previous studies found that the activities of MDHAR and DHAR in kiwifruit with different genotypes were significantly positively correlated with T-AsA and AsA/DHA, which was consistent with the results of this study [28]. This also proves that AsA biosynthesis is not the only factor affecting the AsA maintenance level of the fruit of *A. eriantha*. In addition, in this study, the activity of DHAR2 during the fruit growth period was much higher than that of MDHAR5, which was consistent with the previous results [25]. We speculated that DHAR2 played a more important role in the regeneration cycle pathway of AsA. Studies on tobacco also found that *DHAR* overexpression was more effective in improving AsA content in tobacco than *MDHAR* overexpression [29]. AsA content decreased as the fruit entered the maturity stage, indicating that *MDHAR* and *DHAR* in the AsA regeneration cycle pathway reduced more AsA in the fruit maturity stage, while the oxidation rate of AsA was lower than that of AsA during the maturity stage, resulting in the decrease of AsA content in the fruit.

The accumulation mechanism of AsA varies among different species, AsA synthesis pathways are different in different organs of the same species or at different development stages. Many plants need multiple ways to regulate AsA accumulation. AsA accumulation of jujube fruit is mainly dependent on the synthesis of AsA through L-galactose pathway, the fruit of jujube was reduced by AsA regeneration cycle [30]. AsA in roxburghia fruit is controlled by alternating coexistence of L-galactose pathway and D-galacturonic acid pathway [31]. In the early stage of tomato and grape fruit development, AsA is mainly synthesized by L-galactose pathway, while in the mature stage, AsA accumulation is controlled by D-galacturonic acid pathway [32, 33]. While, the accumulation of AsA was mainly regulated by the L-galactose pathway and the D-galacturonic acid pathway and the AsA regeneration cycle pathway. However, the contribution of L-gullosugar pathway and inositol pathway to AsA accumulation is not clear and needs further study.

## Conclusion

In summary, AsA reached the accumulation peak at DAF110 during the process of fruit growth and development. The activity of GalDH, GalLDH, MDHAR and DHAR in fruit was similar to AsA accumulation trend, and these enzymes were significantly positively correlated with AsA content. It was speculated that GalDH and GalLDH were key enzymes in AsA biosynthesis, while MDHAR and DHAR were key enzymes in AsA regeneration cycle, which together regulated AsA accumulation in fruit. In addition, 23 candidate genes related to AsA biosynthesis and degradation were screened out form RNA-seq. Considering gene expression levels and changes of physiological traits and related enzyme activity, we concluded that the accumulation of AsA depends mainly on the L-galactose pathway, and the D-galacturonic acid pathway and AsA recycling pathway as the secondary pathways, which co-maintain the high AsA content in fruit of *A. eriantha*.

## Methods

### Materials

‘Ganmi 6’ (*A. eriantha*) were grown in the germplasm resources garden of Kiwifruit Research Institute in Fengxin County, Jiangxi Province, China. Fruit samples were collected from 11 stages, which were as follows: 20 days after full bloom (DAF) (S1), 35 DAF (S2), 50 DAF (S3), 65 DAF (S4), 80 DAF (S5), 95 DAF (S6), 110 DAF (S7), 125 DAF (S8), 140 DAF (S9), 155 DAF (S10) and 170 DAF (S11). By 170 DAF (S11), the fruit of ‘Ganmi 6’ have reached harvest maturity (SSC = 6.5% [18]). A total of six vines, similar in size, bearing and receiving sunlight uniformly were used for the experiment and randomly divided into three groups (replicates) with 2 vines in each group. At per developmental stage, about 20 fruit from different directions (east, south, west and north) of each group were collected, and packed and placed in an icebox. Then the fruit were immediately transported to the laboratory, the exocarp and endocarp were removed, only the mesocarp was chopped and immediately frozen with liquid nitrogen, and stored at − 80 °C for later use. Three biological replicates were set for each period.

### Measurement of AsA content and related metabolic enzymes

AsA was determined by previous research [34]. The DHA content and AO enzyme activity were determined by plant DHA content determination kit and the AO assay kit of Solarbio Biotechnology Co., LTD, respectively. The content of T-ASA was calculated according to the following formula: T-ASA (mg/g) = AsA + DHA. GalDH, GalLDH, MDHAR, DHAR, GalUR and APX enzyme activity were determined by previous research [22, 25, 35, 36].

### RNA isolation, library preparation, and sequencing

Total RNA was extracted by Trizol reagent kit (Invitrogen, Carlsbad, CA, USA) according to the manufacturer’s protocol. RNA quality was assessed by an Agilent 2100 Bioanalyzer (Agilent Technologies, Palo Alto, CA, USA) and checked by RNase free agarose gel electrophoresis. After total RNA was extracted, eukaryotic mRNA was enriched by Oligo (dT) beads, while prokaryotic mRNA was enriched by removing rRNA by Ribo-Zero™ Magnetic Kit (Epicentre, Madison, WI, USA). Then the enriched mRNA was fragmented into short fragments by fragmentation buffer and reverse transcribed into cDNA with random primers. Second-strand cDNA was synthesized by DNA polymerase I, RNase H, dNTP and buffer. Then the cDNA fragments were purified with QiaQuick PCR extraction kit (Qiagen, Venlo, The Netherlands), end repaired, poly(A) added, and ligated to Illumina sequencing adapters. The ligation products were size selected by agarose gel electrophoresis, PCR amplified, and sequenced by Illumina novaseq 6000 by Gene Denovo Biotechnology Co. (Guangzhou, China).

### Data processing assembly

Reads obtained from the sequencing machines included raw reads, containing adapters or low quality bases which would affect the following assembly and analysis. Thus, to get high quality clean reads, reads were further filtered by fastp (version 0.18.0) [37]. Transcriptome denovo assembly was carried out with short reads assembling program -Trinity [38]. The data of RNA-seq was been uploaded to NCBI, the ID is PRJNA668457.

### Annotation and classification of gene functions

Basic annotation of unigenes includes protein functional annotation, pathway annotation, COG/KOG functional annotation and Gene Ontology (GO) annotation. To annotate the unigenes, we used BLASTx program (http://www.ncbi.nlm.nih.gov/BLAST/) with an e-value threshold of 1e-5 to NCBI non-redundant protein (Nr) database (http://www.ncbi.nlm.nih.gov), the Swiss-Prot protein database (http://www.expasy.ch/sprot), the Kyoto Encyclopedia of Genes and Genomes (KEGG) database (http://www.genome.jp/kegg), and the COG/KOG database (http://www.ncbi.nlm.nih.gov/COG). Protein functional annotations could then be obtained according to the best alignment results.

### qRT-PCR analysis and cluster analysis

Twenty-three novel transcripts related to ascorbic acid biosynthesis were selected for expression pattern determination. Specific primers were designed by Primer 5.0 (supplementary Table 1). The RNA of fleshed in each period was extracted according to previous research [39]. Then cDNA was synthesized by the TaKaRa Reagent Kit (PremeScript TM RT Reagent Kit with gDNA Eraser, Perfect Real Time). The PCR mixture contained 2.5 μL double distilled water (ddH_2_O), 5 μL SYBR Green I master mix (Asbio Technology, Inc.), 0.1 μM of the forward and reverse primers for each gene, and 1.5 μL cDNA template. The LightCycler® 480 real-time PCR system with a 96-well plate was used to conduct the reaction. The conditions for the PCR amplifications were as follows: 95 °C for 5 min, followed by 45 cycles of 10 s at 95 °C, 20 s at 60 °C, and 20 s at 72 °C. At the end of each experiment, a melt-curve analysis was carried out by the default parameters (5 s at 95 °C and 1 min at 65 °C). The *Actin* (*Actin isoform B*) in the kiwifruit was considered as the control gene for normalization [40]. Three biological replicates were set for every analysis. The relative expressions were calculated by the 2^−ΔΔCt^ method [41], and Microsoft Office Excel 2016 (Microsoft Corporation, Redmond, USA) was used for chart preparation. IBM SPSS Statistics 20 was used for correlation analysis and difference analysis.

## Supplementary Information

**Additional file 1: Supplementary Figure 1.** The length distribution of splicing unigenes. The X-axis is the length of the unigenes, the unit is the bp. The Y-axis is the number of unigenes.

**Additional file 2: Supplementary Figure 2.** Venn diagram of unigenes annotated in four major databases. NR: RefSeq non-redundant proteins; SwissProt: the manually annotated and reviewed section of the UniProt Knowledgebase; KOG: clusters of euKaryotic Orthologous Groups; KEGG: Kyoto Encyclopedia of Genes and Genomes.

**Additional file 3: Supplementary Figure 3.** Gene ontology classification of assembled unigenes. Forty thousand one hundred seven unigenes were assigned to three GO categories with 22 GO terms of biological process, 21 GO terms of cellular component, and 10 GO terms of molecular function. The x-axis represents GO terms belonging to three GO categories and the y-axis indicates the number of genes.

**Additional file 4: Supplementary Figure 4.** KOG functional classification. A total of 29,116 unigenes showed significant similarity to the sequences in KOG databases and were clustered into 25 categories. The x-axis represents the name of 25 groups and the y-axis indicates proportion of genes annotated under the group accounts occupy the total number of genes being annotated.

**Additional file 5: Supplementary Figure 5.** KEGG classification of assembled unigenes. The 31,069 annotated unigenes were assigned to 5 KEGG biochemical pathways: cellular processes, environmental information processing, genetic information processing, metabolism and organism system. The x-axis represents number of genes annotated under the pathway and the y-axis indicates the name of metabolic pathway of KEGG.

**Additional file 6: Supplementary Figure 6.** Volcano Plot of three groups. The X-axis represents the logarithm of the difference multiples of the two samples, and the Y-axis represents the negative log of the FDR of the two samples. The red point refers to the up-regulated genes, the green point refers to the down-regulated genes, and the black point refers to no difference. The criterion for the difference in expression level was FDR < 0.05, and the difference multiple was more than two times. (a) DEGs among S6 vs S7; (b) DEGs among S6 vs S8; (c) DEGs among S7 vs S8.

**Additional file 7: Supplementary Figure 7.** Expression analysis of genes related to the AsA synthesis pathway during the whole fruit developments. L-galactose pathway: a-j, D-galacturonic acid pathway: g and h, L-gullosugar pathway: m, inositol pathway: n and o.

**Additional file 8: Supplementary Figure 8.** Expression analysis of genes related to the AsA cycle pathway during the whole fruit developments.

**Additional file 9: Supplementary Table 1.** Real-time PCR-specific primer sequences.

**Additional file 10: Supplementary Table 2.** Summary statistics of ‘Ganmi 6’ transcriptome assembly. When the cumulative fragment length reaches 50% of the total fragment length (the length of all unigenes), the corresponding length and number of that fragment is the length and number of unigenes N50. The longer the unigenes N50, the smaller the quantity, the better the assembly quality.

**Additional file 11: Supplementary Table 3.** 23 DEGs involved in AsA biosynthesis and circulation.

**Additional file 12: Supplementary Table 4.** Correlation analysis of AsA metabolic related components and their related enzymes and genes.

## Data Availability

All data generated or analyzed during this study were included in this published article. The data of RNA-seq was uploaded to NCBI, ID: PRJNA668457.

## Abbreviations

AsA: Ascorbic acid
PGI: Glucose isomerase
PMI: Phosphomannose isomerase
GME: GDP-D-mannose-3 ‘and 5’ -hetero isomerase
PMM: Phosphoric mannose enzyme
GalDH: L-galactose dehydrogenase
GalLDH: L-galactono-1,4-lactone dehydrogenase
GalUR: D-galacturonic acid reductase
MIOX: Inositol oxygenase
AAO: L-ascorbate oxidase
APX: L-ascorbate peroxidase
GMP: GDP-mannose pyrophosphorylase
GPP: L-Galactose-1-phosphate phosphatase
GGP: GDP-L-galactose-1-phosphate phosphorylase
GuLO: L-gulonolactone oxidase gene
DHAR: Dehydroascorbate reductase
GSSG: Oxidized glutathione
GSH: Reduced glutathione
GR: Glutathione reductase
NADP: Nicotinamide adenine dinucleotide phosphate
NADPH: The reduced form of nicotinamide adenine dinucleotide
GO: Gene ontology
KEGG: Kyoto encyclopedia of genes and genomes
DEGs: Differentially expressed genes
Nr: RefSeq non-redundant proteins
SwissProt: Annotated protein sequence database
KOG: Clusters of orthologous groups for eukaryotic complete genomes

## References

[1] Gallie, Daniel R. L-Ascorbic Acid: A multifunctional molecule supporting plant growth and development. Scientifica. 2013;2013:795964. 10.1155/2013/795964 PMC3820358 24278786

[2] Tang JL, Wu H, Lang BB, Qu XY, Huang CH, Xu XB (2014). Association analysis on leaf and fruit AsA content and SSR markers of wild *Actinidia eriantha*. Acta Horticulturae Sinica.

[3] Wheeler GL, Jones MA, Smirnoff N (1998). The biosynthetic pathway of vitamin C in higher plants. Nature.

[4] Wolucka BA, Montagu MV (2003). GDP-mannose-3′,5′-epimerase forms GDP-L-gulose, a putative intermediate for the de novo biosynthesis of vitamin C in plants. J Biol Chem.

[5] Valpuesta V, Botella MA (2004). Biosynthesis of L-ascorbic acid in plants: new pathways for an old antioxidant. Trends Plant Sci.

[6] Pasqualini S, Batini P, Ederli L, Antonielli M (1999). Responses of the xanthophyll cycle pool and ascorbate-glutathione cycle to ozone stress in two tobacco cultivars. Free Radic Res Commun.

[7] Li J, Liang D, Li MJ, Ma FW (2013). Light and abiotic stresses regulate the expression of GDP-l-galactose phosphorylase and levels of ascorbic acid in two kiwifruit genotypes via light-responsive and stress-induciblecis-elements in their promoters. Planta.

[8] Linster CL, Clarke SG (2008). L-Ascorbate biosynthesis in higher plants: the role of VTC2. Trends Plant Sci.

[9] Maruta T, Yonemitsu M, Yabuta Y, Tamoi M, Ishikawa T, Shigeoka S (2008). Arabidopsis Phosphomannose Isomerase 1, but not Phosphomannose Isomerase 2, is essential for ascorbic acid biosynthesis. J Biol Chem.

[10] Qian WQ, Chunmei YU, Qin HJ, Liu X, Zhang AM, Johansen EI, Wang DW (2010). Molecular and functional analysis of phosphomannomutase (PMM) from higher plants and genetic evidence for the involvement of PMM in ascorbic acid biosynthesis in Arabidopsis and *Nicotiana benthamiana*. Plant J Cell Mol Biol.

[11] Tao JJ, Wu H, Li ZY, Huang CH, Xu XB. Molecular evolution of GDP-D-mannose epimerase (GME), a key gene in plant ascorbic acid biosynthesis. Front Plant Sci. 2018;9:1293. 10.3389/fpls.2018.01293 PMC6132023 30233629

[12] Zhang CJ, Liu JX, Zhang YY, Cai XF, Gong PJ, Zhang JH, Wang TT, Li HX, Ye ZB (2011). Overexpression of *SlGMEs* leads to ascorbate accumulation with enhanced oxidative stress, cold, and salt tolerance in tomato. Plant Cell Rep.

[13] Li MJ, Gao J, Ma FW, Liang D, Hou CM (2010). Relationship between expressions of GalDH and GalLDH and ascorbate content in apple fruits. Sci Agric Sin.

[14] Li MJ (2009). Physiological and molecular mechanisms of ascorbic acid formation and accumulation in apple and kiwifruit. Doctor.

[15] Leferink GHN, Willy BAM, Willem BH (2008). L-Galactono-gamma- lactone dehydrogenase from Arabidopsis thaliana, a flavoprotein involved in vitamin C biosynthesis. FEBS J.

[16] Li MJ, Liu J, Liang D, Guo CM, Ma FW (2011). The relationship between GalUR expression and ascorbate accumulation in kiwifruit. Acta Horticulturae Sinica.

[17] Haroldsen VM, Chi-Ham CL, Kulkarni S, Lorence A, Bennett AB (2011). Constitutively expressed *DHAR* and *MDHAR* influence fruit, but not foliar ascorbate levels in tomato. Plant Physiol Biochem.

[18] Liao GL, He YQ, Li XS, Zhong M, Huang CH, Yi SY, Liu Q, Xu XB. Effects of bagging on fruit flavor quality and related gene expression of AsA synthesis in *Actinidia eriantha*. Sci Hortic. 2019;256:108511.

[19] Zhong CH, Zhang P, Jiang ZW, Wang SM, Han F, Xu LY, Huang HW (2011). Dynamic changes of carbohydrate and vitamin c in fruits of *Actinidia chinensis* and *A. eriantha* during growing season. J Wuhan Bot Res.

[20] Voxeur A, Gilbert L, Rihouey C, Driouich A, Rothan C, Baldet P, Lerouge P (2011). Silencing of the GDP-d-mannose 3,5-epimerase affects the structure and cross-linking of the pectic polysaccharide rhamnogalacturonan ii and plant growth in tomato. J Biol Chem.

[21] 1Li J, Li MJ, Liang D, Ma FW, Lei YS. Comparison of expression pattern, genomic structure, and promoter analysis of the gene encoding GDP-l-galactose phosphorylase from two *Actinidia* species. Sci Hortic. 2014;169(02):206–13.

[22] Zhong Y (2017). Study on ascorbic acid metabolism of Actinidia eriantha cv. ‘White’ during storage. Master.

[23] Liu YH, Yu L, Wang RZ (2011). Level of ascorbic acid in transgenic rice for l-galactono-1,4-lactone dehydrogenase overexpressing or suppressed is associated with plant growth and seed set. Acta Physiol Plant.

[24] Dong YM (2011). Clonging and functional analysis of L-galactono-1,4-lactone dehydrogenase gene in potato. Doctor.

[25] Zhou CH (2015). Research on fruit quality and metabolism characteristics of *Actindia eriantha* benth cv. ‘White’ during storage. Master.

[26] Amaya I, Osorio S, Martinez E, LimalogIlva V, Valpuesta V (2015). Increased antioxidant capacity in tomato by ectopic expression of the strawberry D-galacturonate reductase gene. Biotechnol J.

[27] Eduardo C, Iraida A, José S, Miguel B, Victoriano V (2011). Regulation of L-ascorbic acid content in strawberry fruits. J Exp Bot.

[28] Yuan YL, Tong XL, Hou CM, Ma FW, Li MJ (2016). Difference of ascorbic acid synthesis and metabolism in different genotypes of kiwifruit. Plant Physiol Commun.

[29] Yin LN, Wang SW, Eltayeb AE, Uddin M, Yamamoto Y, Tsuji W, Takeuchi Y, Tanaka K (2010). Overexpression of dehydroascorbate reductase, but not monodehydroascorbate reductase, confers tolerance to aluminum stress in transgenic tobacco. Planta.

[30] Chen YY (2015). Synthesis and metabolism of L-ascorbic acid in Chinese jujube and wild jujube. Master.

[31] An HM, Chen LG, Fan WG, Liu QL (2005). Relationship between ascorbic acid accumulation and related enzyme activities in fruit of rosa roxburghii tratt. J Plant Physiol Mol Biol.

[32] Melino VJ, Soole KL, Ford CM (2009). Ascorbate metabolism and the developmental demand for tartaric and oxalic acids in ripening grape berries. BMC Plant Biol.

[33] Badejo AA, Wada K, Gao YS, Maruta T, Sawa Y, Shigeoka S, Ishikawa T (2012). Translocation and the alternative D-galacturonate pathway contribute to increasing the ascorbate level in ripening tomato fruits together with the D-mannose/L-galactose pathway. J Exp Bot.

[34] Liao GL, Xu XB, Zhong M, Huang CH, Tu GQ, Li BM, Tao JJ, Qu XY, Zhao SG, Leng JH (2019). A novel mid-maturing cultivar with high dry matter content from seedlings of ‘Jinfeng’ kiwifruit (*Actinidia chinensis*). Eur J Hortic Sci.

[35] Gatzek S, Wheeler GL, Smirnoff N (2002). Antisense suppression of L-galactose dehydrogenase in *Arabidopsis thaliana* provides evidence for its role in ascorbate synthesis and reveals light modulated L-galactose synthesis. Plant J.

[36] Kazuko B, Ishikawa S, Nishikawa M, Mizuno H, Yamamoto T (1995). Purification and properties of L-Galactono- γ-lactone dehydrogenase, a key enzyme for ascorbic acid biosynthesis, from sweet potato roots. J Biochem.

[37] Chen SF, Zhou YQ, Chen YR, Gu J (2018). Fastp: an ultra-fast all-in-one FASTQ preprocessor. Bioinformatics.

[38] Grabherr MG, Haas BJ, Yassour M, Levin JZ, Thompson DA, Amit I, Adiconis X, Fan L, Raychowdhury R, Zeng Q (2011). Full-length transcriptome assembly from RNA-Seq data without a reference genome. Nat Biotechnol.

[39] Liao GL, Liu Q, Li YQ, Zhong M, Huang CH, Jia DF, Xu XB. Identification and expression profiling analysis of ascorbate peroxidase gene family in *Actinidia chinensis* (Hongyang). J Plant Res. 2020;133:715–26. 10.1007/s10265-020-01206-y 32506283

[40] Zhang HQ, Xie M, Xiao JP, Zhou LQ, Song GH (2015). Screening of reference genes for real-time quantitative PCR in kiwifruit. Acta Agric Zhejiangensis.

[41] Vandesompele J, Preter DP, Pattyn F, Poppe B, Roy NV, Paepe AD, Speleman F (2002). Accurate normalization of real-time quantitative RT-PCR data by geometric averaging of multiple internal control genes. Genome Biol.

