# Supplementary figures and images for: Three metabolic pathways are responsible for the accumulation and maintenance of high AsA content in kiwifruit (Actinidia eriantha)
Source: BMC Genomics. 2021 Jan 6;22:13. doi: 10.1186/s12864-020-07311-5 (PMC7788711; doi:10.1186/s12864-020-07311-5)

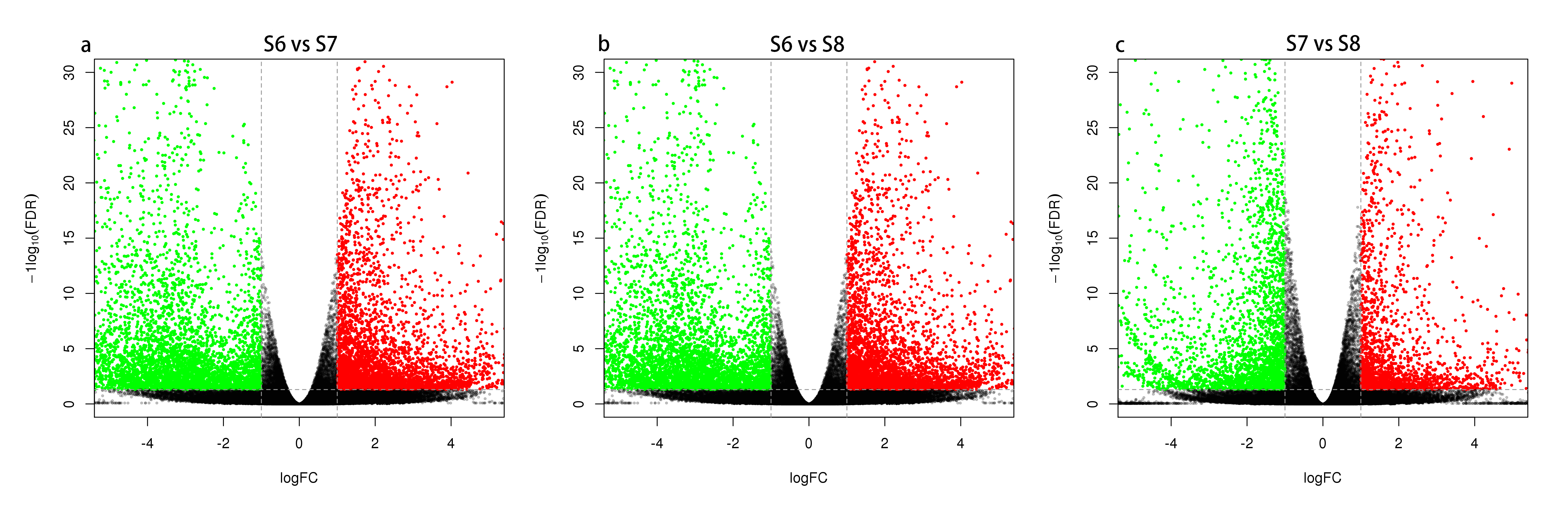

Supplement: Supplementary file 6 — Additional file 6: Supplementary Figure 6. Volcano Plot of three groups. The X-axis represents the logarithm of the difference multiples of the two samples, and the Y-axis represents the negative log of the FDR of the two samples. The red point refers to the up-regulated genes, the green point refers to the down-regulated genes, and the black point refers to no difference. The criterion for the difference in expression level was FDR < 0.05, and the difference multiple was more than two times. (a) DEGs among S6 vs S7; (b) DEGs among S6 vs S8; (c) DEGs among S7 vs S8. [file 12864_2020_7311_MOESM6_ESM.tif]
