# Supplementary material for: Three metabolic pathways are responsible for the accumulation and maintenance of high AsA content in kiwifruit (Actinidia eriantha)
Source: BMC Genomics. 2021 Jan 6;22:13. doi: 10.1186/s12864-020-07311-5 (PMC7788711; doi:10.1186/s12864-020-07311-5)
Supplement: Supplementary file 9 — Additional file 9: Supplementary Table 1. Real-time PCR-specific primer sequences. [file 12864_2020_7311_MOESM9_ESM.docx]

Supplementary table1. Real-time PCR-specific primer sequences.

| Gene ID | Gene annotation | Upstream primers (5’-3’) | Downstream primers (5’-3’) |
| --- | --- | --- | --- |
| *Unigene0079058* | *PGI1* | CGCAAAATCTGTCGCTCGAG | CAGCCAGTCGACGTATCTCC |
| *Unigene0071745* | *PMI2* | CCCGGCCCCTCTATTTTTGT | ACCTGCTATTAACGCCAGCT |
| *Unigene0075397* | *PMI1* | CCTTCGGAGTTAAGCCAGCA | ATGTGGGTGTCCTAGCAAGC |
| *Unigene0049528* | *PMM* | GGAAGCCTGGAGTGATTGCT | CTGGTGCATTCAGGGGTGAT |
| *Unigene0014022* | *GME* | GCAATGTGCGAGGCAATGAA | TGGGAAGCACCTGTGAATCT |
| *Unigene0026576* | *GGP1* | TCTCAGCATAGCACTGTGGTAAG | GAAGGTGGAAACTCTCTGGAAG |
| *Unigene0073983* | *GGP2* | GCTGCGCAATGAAACCATACT | ACTCAACCTCTCCATCTTAGTCA |
| *Unigene0046069* | *GPP1* | TTGGAAACATGCTCCGCAAC | AGCTGCTCGTGTCTTTGTTG |
| *Unigene0014860* | *GalDH* | TCGGTGTTTTTGGTGTGTCC | CCCAGTACTTTCTCCGACAATG |
| *Unigene0048250* | *GalLDH* | TGGTGAATTTGGCCCTCATG | ACGCGAAATTCTGCAAGGTG |
| *Unigene0032122* | *GuLO6* | TGGCATTTGAGGGAATGAGC | ACAATGTTCACCCCTCCATCTC |
| *Unigene0014549* | *GalUR1* | TGGAGTTGTGCAAGGCAAAG | TGGCAATCTCCTCAAGGACATC |
| *Unigene0050219* | *GalUR2* | TATGAAGTCAGTGTGGGAAGCC | TGATGGCTGGTGGGATTTTG |
| *Unigene0022038* | *MIOX1* | TGTTGGACGGTGGTTTTGTG | TCTCAACAACTCCTTGCCTGAG |
| *Unigene0059542* | *MIOX2* | TCGACCGTCTTTTGCCTTTC | TGCAGTACCAAAACCTGTGC |
| *Unigene0071888* | *MDHAR5* | TCGTACGTGTGAGTTTGTGC | TGATCGCCTTGCAAGAGTTG |
| *Unigene0047736* | *DHAR2* | AGCCCGGGTTTTGACAAATG | AGATTGCGTTGGGGCATTAC |
| *Unigene0052046* | *DHAR3* | TTCGTTGTCGCGTGCTATTG | AAACCAACGGACACATCTGC |
| *Unigene0052112* | *APX2* | TTTACACCCATTGCCTGTGC | AATCCAGAACGCTCCTTGTG |
| *Unigene0055048* | *APX1* | TCCGCTTATGCTCCGTCTC | GAAACTGTTCCTTGATGGGCTC |
| *Unigene0064494* | *APX5* | TTCACAGCTTTCGCATCTGC | TGCAGCATTAGCACGGTATC |
| *Unigene0064656* | *APX3* | AGGTAAGTCAGGACCACCACAG | CTCTCCGAATTAGGGTTCACTC |
| *Unigene0057469* | *AAO* | TGGGCCGTCAACAACATTTC | TGTTCAAATGCGCCGTTCAG |
| *Actin* | | GTGCTCAGTGGTGGTTCAA | GACGCTGTATTTCCTCTCAG |
