# Supplementary material for: Three metabolic pathways are responsible for the accumulation and maintenance of high AsA content in kiwifruit (Actinidia eriantha)
Source: BMC Genomics. 2021 Jan 6;22:13. doi: 10.1186/s12864-020-07311-5 (PMC7788711; doi:10.1186/s12864-020-07311-5)
Supplement: Supplementary file 10 — Additional file 10: Supplementary Table 2. Summary statistics of ‘Ganmi 6’ transcriptome assembly. When the cumulative fragment length reaches 50% of the total fragment length (the length of all unigenes), the corresponding length and number of that fragment is the length and number of unigenes N50. The longer the unigenes N50, the smaller the quantity, the better the assembly quality. [file 12864_2020_7311_MOESM10_ESM.docx]

Supplementary table 2. Summary statistics of ‘Ganmi 6’ transcriptome assembly. When the cumulative fragment length reaches 50% of the total fragment length (the length of all unigenes), the corresponding length and number of that fragment is the length and number of unigenes N50. The longer the unigenes N50, the smaller the quantity, the better the assembly quality.

| Genes number | GC(%) | N50 | Max length  /bp | Min length  /bp | Average length  bp | Total assembled bases  /bp |
| --- | --- | --- | --- | --- | --- | --- |
| 98656 | 41.30 | 1609 | 16709 | 201 | 932 | 91956584 |
