# Supplementary material for: Three metabolic pathways are responsible for the accumulation and maintenance of high AsA content in kiwifruit (Actinidia eriantha)
Source: BMC Genomics. 2021 Jan 6;22:13. doi: 10.1186/s12864-020-07311-5 (PMC7788711; doi:10.1186/s12864-020-07311-5)
Supplement: Supplementary file 11 — Additional file 11: Supplementary Table 3. 23 DEGs involved in AsA biosynthesis and circulation. [file 12864_2020_7311_MOESM11_ESM.docx]

Supplementary table 3. 23 DEGs involved in AsA biosynthesis and circulation.

| Gene ID | Map in ‘Hongyang’ genome | Gene annotation | Pathway |
| --- | --- | --- | --- |
| Unigene0079058 | Achn087691 | *PGI1* | L-galactose pathway |
| Unigene0071745 | Achn292661 | *PMI2* |  |
| Unigene0075397 | Achn330131 | *PMI1* |  |
| Unigene0049528 | Achn302501 | *PMM* |  |
| Unigene0014022 | Achn054171 | *GME* |  |
| Unigene0026576 | Achn339231 | *GGP1* |  |
| Unigene0073983 | Achn155031 | *GGP2* |  |
| Unigene0046069 | Achn311491 | *GPP1* |  |
| Unigene0014860 | Achn334011 | *GalDH* |  |
| Unigene0048250 | Achn136491 | *GalLDH* |  |
| Unigene0032122 | Achn346051 | *GuLO6* | inositol pathway |
| Unigene0022038 | Achn115461 | *MIOX1* |  |
| Unigene0059542 | Achn310731 | *MIOX2* |  |
| Unigene0014549 | Achn110361 | *GalUR1* | D-galacturonic acid pathway |
| Unigene0050219 | Achn022001 | *GalUR2* |  |
| Unigene0071888 | Achn132811 | *MDHAR5* | AsA circulation pathway |
| Unigene0047736 | Achn278191 | *DHAR2* |  |
| Unigene0052046 | Achn007991 | *DHAR3* |  |
| Unigene0052112 | Achn059971 | *APX2* |  |
| Unigene0055048 | Achn315041 | *APX1* |  |
| Unigene0064494 | Achn187071 | *APX5* |  |
| Unigene0064656 | Achn340531 | *APX3* |  |
| Unigene0057469 | Achn228041 | *AAO* |  |
