# Supplementary material for: Three metabolic pathways are responsible for the accumulation and maintenance of high AsA content in kiwifruit (Actinidia eriantha)
Source: BMC Genomics. 2021 Jan 6;22:13. doi: 10.1186/s12864-020-07311-5 (PMC7788711; doi:10.1186/s12864-020-07311-5)
Supplement: Supplementary file 12 — Additional file 12: Supplementary Table 4. Correlation analysis of AsA metabolic related components and their related enzymes and genes. [file 12864_2020_7311_MOESM12_ESM.docx]

Supplementary table 4. Correlation analysis of AsA metabolic related components and their related enzymes and genes.

|  | AsA | DHA | T-AsA | AsA/DHA | GalDH | GalLDH | GalUR | MDHAR | DHAR | APX | AAO | *PGI1* | *PMI2* | *PMI1* | *PMM* | *GME* | *GGP1* | *GGP2* |
| --- | --- | --- | --- | --- | --- | --- | --- | --- | --- | --- | --- | --- | --- | --- | --- | --- | --- | --- |
| AsA | 1 |  |  |  |  |  |  |  |  |  |  |  |  |  |  |  |  |  |
| DHA | -0.65* | 1 |  |  |  |  |  |  |  |  |  |  |  |  |  |  |  |  |
| T-AsA | 1.00** | -0.59* | 1 |  |  |  |  |  |  |  |  |  |  |  |  |  |  |  |
| AsA/DHA | 0.89** | -0.85** | 0.85** | 1 |  |  |  |  |  |  |  |  |  |  |  |  |  |  |
| GalDH | 0.73** | -0.29 | 0.74** | 0.56 | 1 |  |  |  |  |  |  |  |  |  |  |  |  |  |
| GalLDH | 0.73** | -0.34 | 0.74** | 0.55 | 0.47 | 1 |  |  |  |  |  |  |  |  |  |  |  |  |
| GalUR | 0.2 | -0.47 | 0.17 | 0.31 | 0.03 | 0.3 | 1 |  |  |  |  |  |  |  |  |  |  |  |
| MDHAR | 0.68* | -0.61* | 0.66* | 0.64* | 0.53 | 0.33 | -0.09 | 1 |  |  |  |  |  |  |  |  |  |  |
| DHAR | 0.89** | -0.60* | 0.88** | 0.74** | 0.66* | 0.82** | 0.23 | 0.55 | 1 |  |  |  |  |  |  |  |  |  |
| APX | -0.04 | 0.29 | -0.01 | -0.1 | 0.08 | -0.24 | 0.1 | -0.04 | -0.37 | 1 |  |  |  |  |  |  |  |  |
| AAO | 0.18 | 0.09 | 0.2 | 0.01 | -0.03 | 0.22 | 0.13 | -0.12 | 0 | 0.21 | 1 |  |  |  |  |  |  |  |
| *PGI1* | -0.15 | 0.06 | -0.15 | -0.16 | -0.01 | 0.29 | 0.51 | -0.24 | 0.08 | -0.19 | 0.13 | 1 |  |  |  |  |  |  |
| *PMI2* | -0.01 | -0.08 | -0.02 | 0.03 | 0.04 | 0.02 | 0.15 | -0.09 | 0.29 | -0.26 | -0.64* | 0.23 | 1 |  |  |  |  |  |
| *PMI1* | -0.04 | -0.06 | -0.05 | 0.07 | -0.03 | 0.14 | -0.03 | 0.17 | 0.15 | -0.16 | -0.59* | 0.24 | 0.72** | 1 |  |  |  |  |
| *PMM* | 0.16 | -0.04 | 0.17 | 0.01 | 0.22 | 0.44 | 0.29 | 0.12 | 0.47 | -0.15 | -0.34 | 0.57 | 0.75** | 0.66* | 1 |  |  |  |
| *GME* | 0.67* | -0.37 | 0.67* | 0.47 | 0.47 | .651* | 0.51 | 0.09 | 0.76** | -0.19 | 0.36 | 0.26 | 0.25 | -0.08 | 0.39 | 1 |  |  |
| *GGP1* | 0.61* | -0.31 | 0.61* | 0.46 | 0.38 | 0.52 | 0.43 | 0.09 | 0.65* | 0.06 | 0.36 | 0.3 | 0.38 | 0.13 | 0.51 | 0.85** | 1 |  |
| *GGP2* | 0.43 | -0.59* | 0.39 | 0.52 | 0.28 | 0.26 | 0.21 | 0.84** | 0.26 | 0.13 | -0.17 | -0.05 | -0.21 | 0.2 | 0.07 | -0.13 | -0.13 | 1 |
| *GPP1* | 0.14 | -0.01 | 0.15 | -0.02 | 0.18 | 0.45 | 0.26 | 0.13 | 0.45 | -0.17 | -0.27 | 0.61* | 0.67* | .623* | 0.99** | 0.35 | 0.48 | 0.09 |
| *GalDH* | 0.45 | -0.26 | 0.45 | 0.36 | 0.59* | 0.45 | 0.33 | 0.2 | 0.67* | -0.16 | -0.43 | 0.4 | 0.76** | 0.48 | 0.82** | 0.55 | 0.59* | 0.05 |
| *GalLDH* | 0.58* | -0.48 | 0.57 | 0.52 | 0.52 | 0.64* | 0.44 | 0.39 | 0.76** | -0.25 | -0.16 | 0.57 | 0.48 | 0.35 | 0.76** | 0.55 | 0.62* | 0.3 |
| *GuLO6* | 0.3 | -0.22 | 0.29 | 0.25 | 0.35 | 0.39 | 0.29 | 0.2 | 0.55 | -0.17 | -0.49 | 0.49 | 0.84** | 0.70* | 0.92** | 0.39 | 0.54 | 0.12 |
| *GalUR1* | 0.87** | -0.55 | 0.86** | 0.84** | 0.71** | 0.73** | 0.21 | 0.53 | 0.77** | -0.13 | 0.23 | 0.18 | -0.05 | 0.04 | 0.14 | 0.58* | 0.55 | 0.37 |
| *GalUR2* | 0.29 | 0.06 | 0.31 | 0.02 | 0.27 | .626* | 0.19 | 0.29 | 0.47 | 0.04 | 0 | 0.51 | 0.31 | 0.48 | 0.85** | 0.34 | 0.44 | 0.28 |
| *MIOX1* | -0.07 | 0.25 | -0.05 | -0.12 | 0.34 | 0.1 | 0.08 | -0.1 | 0.08 | 0.25 | -0.34 | 0.46 | 0.56 | 0.60* | 0.70* | 0.05 | 0.3 | -0.08 |
| *MIOX2* | 0.43 | 0 | 0.46 | 0.14 | 0.38 | .630* | 0.09 | 0.46 | 0.49 | 0.18 | 0.11 | 0.34 | 0.15 | 0.38 | 0.71** | 0.32 | 0.45 | 0.4 |
| *MDHAR5* | 0.63* | -0.51 | 0.61* | 0.56 | 0.59* | 0.56 | 0.29 | 0.58* | 0.76** | -0.12 | -0.34 | 0.31 | 0.59* | 0.58* | 0.80** | 0.47 | 0.59* | 0.44 |
| *DHAR2* | 0.60* | -0.42 | 0.60* | 0.47 | 0.58* | 0.42 | 0.27 | 0.38 | 0.77** | -0.17 | -0.28 | 0.2 | 0.70* | 0.36 | 0.74** | 0.64* | 0.72** | 0.09 |
| *DHAR3* | -0.16 | -0.01 | -0.17 | -0.17 | -0.1 | 0.17 | 0.34 | -0.28 | 0.22 | -0.42 | -0.25 | 0.76** | 0.64* | 0.35 | 0.74** | 0.24 | 0.34 | -0.26 |
| *APX2* | 0.43 | -0.29 | 0.42 | 0.3 | 0.48 | 0.51 | 0.34 | 0.19 | 0.70* | -0.41 | -0.23 | 0.56 | 0.57 | 0.22 | 0.74** | 0.58* | 0.53 | 0.03 |
| *APX1* | 0.09 | -0.09 | 0.08 | 0.13 | 0.23 | -0.45 | 0.05 | 0.03 | -0.13 | 0.43 | 0.12 | -0.29 | 0.12 | -0.06 | -0.21 | 0.19 | 0.32 | -0.13 |
| *APX5* | 0.27 | -0.14 | 0.28 | 0.18 | 0.56 | 0.24 | 0.35 | 0.19 | 0.3 | -0.11 | -0.11 | 0.27 | 0.06 | -0.09 | 0.15 | 0.41 | 0.06 | 0.2 |
| *APX3* | -0.27 | -0.04 | -0.29 | -0.25 | -0.1 | -0.24 | 0.15 | 0.11 | -0.07 | -0.15 | -0.21 | 0.56 | 0.5 | 0.49 | 0.59* | -0.02 | 0.19 | 0.11 |
| *AAO* | 0.59* | -0.28 | 0.60* | 0.43 | 0.48 | 0.74** | 0.39 | 0.04 | 0.73** | -0.4 | 0.33 | 0.36 | 0.15 | -0.03 | 0.31 | 0.90** | .652* | -0.15 |

Continued

|  | *GPP1* | *GalDH* | *GalLDH* | *GuLO6* | *GalUR1* | *GalUR2* | *MIOX1* | *MIOX2* | *MDHAR5* | *DHAR2* | *DHAR3* | *APX2* | *APX1* | *APX5* | *APX3* | *AAO* |
| --- | --- | --- | --- | --- | --- | --- | --- | --- | --- | --- | --- | --- | --- | --- | --- | --- |
| AsA |  |  |  |  |  |  |  |  |  |  |  |  |  |  |  |  |
| DHA |  |  |  |  |  |  |  |  |  |  |  |  |  |  |  |  |
| T-AsA |  |  |  |  |  |  |  |  |  |  |  |  |  |  |  |  |
| AsA/DHA |  |  |  |  |  |  |  |  |  |  |  |  |  |  |  |  |
| GalDH |  |  |  |  |  |  |  |  |  |  |  |  |  |  |  |  |
| GalLDH |  |  |  |  |  |  |  |  |  |  |  |  |  |  |  |  |
| GalUR |  |  |  |  |  |  |  |  |  |  |  |  |  |  |  |  |
| MDHAR |  |  |  |  |  |  |  |  |  |  |  |  |  |  |  |  |
| DHAR |  |  |  |  |  |  |  |  |  |  |  |  |  |  |  |  |
| APX |  |  |  |  |  |  |  |  |  |  |  |  |  |  |  |  |
| AAO |  |  |  |  |  |  |  |  |  |  |  |  |  |  |  |  |
| *PGI1* |  |  |  |  |  |  |  |  |  |  |  |  |  |  |  |  |
| *PMI2* |  |  |  |  |  |  |  |  |  |  |  |  |  |  |  |  |
| *PMI1* |  |  |  |  |  |  |  |  |  |  |  |  |  |  |  |  |
| *PMM* |  |  |  |  |  |  |  |  |  |  |  |  |  |  |  |  |
| *GME* |  |  |  |  |  |  |  |  |  |  |  |  |  |  |  |  |
| *GGP1* |  |  |  |  |  |  |  |  |  |  |  |  |  |  |  |  |
| *GGP2* |  |  |  |  |  |  |  |  |  |  |  |  |  |  |  |  |
| *GPP1* | 1 |  |  |  |  |  |  |  |  |  |  |  |  |  |  |  |
| *GalDH* | 0.76** | 1 |  |  |  |  |  |  |  |  |  |  |  |  |  |  |
| *GalLDH* | 0.77** | 0.84** | 1 |  |  |  |  |  |  |  |  |  |  |  |  |  |
| *GuLO6* | 0.89** | 0.93** | 0.84** | 1 |  |  |  |  |  |  |  |  |  |  |  |  |
| *GalUR1* | 0.13 | 0.44 | 0.63* | 0.33 | 1 |  |  |  |  |  |  |  |  |  |  |  |
| *GalUR2* | 0.88** | 0.55 | 0.65* | 0.67* | 0.26 | 1 |  |  |  |  |  |  |  |  |  |  |
| *MIOX1* | 0.67* | 0.65* | 0.46 | 0.70* | 0.05 | 0.56 | 1 |  |  |  |  |  |  |  |  |  |
| *MIOX2* | 0.75** | 0.45 | 0.60* | 0.55 | 0.35 | 0.96** | 0.47 | 1 |  |  |  |  |  |  |  |  |
| *MDHAR5* | 0.77** | 0.86** | 0.89** | 0.88** | 0.56 | 0.69* | 0.56 | 0.68* | 1 |  |  |  |  |  |  |  |
| *DHAR2* | 0.70* | 0.91** | 0.82** | 0.84** | 0.46 | 0.51 | 0.46 | 0.48 | 0.89** | 1 |  |  |  |  |  |  |
| *DHAR3* | 0.76** | 0.61* | 0.66* | 0.71* | -0.03 | 0.45 | 0.49 | 0.24 | 0.44 | 0.5 | 1 |  |  |  |  |  |
| *APX2* | 0.74** | 0.88** | 0.91** | 0.81** | 0.47 | 0.54 | 0.38 | 0.42 | 0.74** | 0.82** | 0.75** | 1 |  |  |  |  |
| *APX1* | -0.28 | 0.03 | -0.23 | -0.08 | 0 | -0.35 | 0.11 | -0.25 | 0 | 0.17 | -0.35 | -0.23 | 1 |  |  |  |
| *APX5* | 0.1 | 0.43 | 0.28 | 0.22 | 0.36 | 0.11 | 0.04 | 0.06 | 0.24 | 0.26 | 0.01 | 0.44 | 0.11 | 1 |  |  |
| *APX3* | 0.61* | 0.35 | 0.37 | 0.52 | -0.2 | 0.4 | 0.48 | 0.29 | 0.43 | 0.37 | 0.60* | 0.36 | 0.17 | 0.01 | 1 |  |
| *AAO* | 0.28 | 0.49 | 0.5 | 0.32 | .664* | 0.3 | 0 | 0.24 | 0.37 | 0.46 | 0.22 | 0.56 | -0.03 | 0.54 | -0.12 | 1 |
